# Forced to move on: An interview study with survivors who have lost a relative to suicide

**DOI:** 10.1111/ppc.13049

**Published:** 2022-02-11

**Authors:** Sally Hultsjö, Säidi M. Ovox, Caroline Olofsson, Mohammad Bazzi, Rikard Wärdig

**Affiliations:** ^1^ Department of Psychiatry Ryhov County Hospital Jönköping Sweden; ^2^ Division of Nursing and Reproductive Health, Department of Health, Medicine and Caring Sciences Linköping University Linköping Sweden; ^3^ Department of Psychiatrics Motala Hospital Motala Sweden

**Keywords:** aftermath, attitudes, experiences, prevention, suicide, survivors

## Abstract

**Purpose:**

To explore the loss of a relative due to suicide.

**Design and Methods:**

Ten survivors of relatives who had taken their lives were interviewed. Data was analyzed from a phenomenological perspective.

**Findings:**

The survivors described how they witnessed the darkness that took over their relative's lifeworld. During the time before and after the suicide, the survivor felt barred from having a role in the relative's care.

**Practice Implications:**

Understanding and exploring implicit dynamics, such as “a feeling of darkness taking over,” “a sense of relief,” or “putting on a mask” could be important for developing person‐centered suicide care.

## 1 INTRODUCTION

Suicide is a serious public health problem, and almost 800,000 people die every year due to suicide World Health Organization (2019). Every suicide is a tragedy with negative and long‐term effects on people connected to the deceased, estimated to number approximately 60 individuals per suicide (Feigelman et al., 2018; World Health Organization, 2021). Living closely with a person in a suicidal process is an all‐consuming and life‐changing process (Juel et al., 2021; Sellin et al., 2017), and becoming a survivor of suicide loss (survivor) is associated with a variety of negative health impacts as well as an increased risk of suicidality (Mogensen et al., 2016). The process of understanding why the suicide occurred is a challenging process for survivors (Dransart, 2017). Even for those who anticipate the suicide in advance, the act comes as a shock. Loss related to suicide is complex, as the suicide can be perceived as shameful and stigmatic (Kõlves et al., 2019; Entilli et al., 2021), while at the same time bringing other feelings, for example, feelings of relief (Pitman et al., 2016; Maple et al., 2007). Feelings of guilt, denial and blame are common (Peters et al., 2016a), which may in turn form a crucial element of a survivor's own suicidal process (Sugrue et al., 2014).

Guilt and grief due to suicide also lead to more isolation than does grief resulting from other causes (Sveen & Walby, 2008; Maple et al., 2018; Peters et al., 2016b), since natural death is less subject to negative reactions from others (Chapple et al., 2015). Social isolation can slow down the grieving process (Levi‐Belz & Lev‐Ari, 2019a, 2019b), and survivors often experience a “prolonged sense of grief” (Shear, 2015) that includes a range of social, mental as well as physical stress‐related reactions and symptoms (Erlangsen et al., 2017; Eckholdt et al., 2018; Peters et al., 2016a).

Although most survivors want to talk to others about the event (Walker, 2017), and social support is found to be helpful (Asare‐Doku et al., 2017), many survivors never encounter such support Shallcross Lam (2014). This social isolation itself creates a further barrier to seeking social support (McMenamy et al., 2008). Survivors highlight the benefit of professional support, while at the same time voicing opinions about the inadequacy of methods by which this support is made available (McMenamy et al., 2008; Nic an Fhailí et al., 2016) and delivered (Lee et al., 2017).

Mental health care has a responsibility to identify and offer effective interventions for those at risk of suicide (Health Care Act 2017, Sweden), and it is valuable for relatives to be involved in their care (Skundberg‐Kletthagen et al., 2014). Yet survivors' experience is that they are not included. Neither do they feel they receive support after the suicide (Andershed et al., 2017). Survivors possess significant expertise and knowledge, both explicit and implicit in nature (Hultsjö et al., 2018; Asare‐Doku et al., 2017). Exploring this knowledge can lead to new ways of understanding the progression of a suicidal process, as well as a deeper understanding of the survivors' own processes, to enable support to be tailored towards survivors. This article explores survivors' experiences of both living through a suicidal progression with their loved one and becoming a suicide survivor, to understand the lived experience of witnessing a suicidal progression. This understanding can increase knowledge about preventive suicide care.

## 2 DESIGN AND METHODS

### 2.1 Design

A phenomenological lifeworld perspective was chosen as it was considered appropriate for capturing the unique experience of the participants and generating new knowledge of the phenomenon (Dahlberg et al., 2008). Fundamental in the lifeworld perspective is to adapt a reflective attitude throughout the research process to discover, describe and clarify the meanings that are based on people's specific and complex life worlds. In the life world perspective, the importance of openness and compliance with the human lived world is emphasized. Everything a person experiences and feels belongs to the lived experience. As a researcher, it is about being open to the unique and meeting a person as unconditionally as possible and at the same time being aware that you can never be completely unconditional. All people carry a pre‐understanding, an understanding or knowledge that makes it possible to understand and relate to the world we live in. In a research process, however, the pre‐understanding must be bridle so that the unpredictable can be captured (Dahlberg 2006; Dahlberg & Dahlberg, 2020). The pre‐understanding otherwise risks becoming an obstacle in such a way that it colors what emerges in the research, which leads to a misleading result. In scientific contexts, the researcher should not be too quick to decide what emerges and stop and ask him/herself what it is and reflect on the understanding that has emerged. By bridle personal ideas and knowledge, an opportunity is created for the phenomenon with its meanings to be revealed as unaffected as possible (Dahlberg et al., 2008). By openly taking part of the phenomenon as described in the relatives' stories with help of a phenomenological lifeworld perspective the essential meanings and the purpose of the study was considered to be captured.

### 2.2 Sample and setting

A convenience sample was used (Polit and Beck, 2016). The inclusion criteria were that the participants should be over 18 years of age and define themselves as survivors who had lost someone to suicide. This definition enabled the participation of both family members and friends. Participation required that the individuals regarded themselves as being in a stable mental state and that the suicide had occurred at least one year previously.

The chairman of the Swedish Association for Suicide Prevention and Survivor Support (SPES) was contacted and received an information letter about the study. After approval, the chairman was asked to provide information to survivors who had lost a relative to suicide. The authors were also invited to a meeting held for survivors. During this meeting, the authors (CO and MB) spoke about the study and gave written information to those who showed interest. Those willing to participate contacted either the authors or the SPES chairman on their own initiative via email or telephone. Interested participants were called by the authors, and the time and place for the interview was decided. Ten survivors, of which nine were women, participated in the study. The participants had different relationships to the person that had committed suicide and included six mothers, one father, one daughter, one mother‐in‐law and one wife. They were aged between 34 and 77 years, (median = 59 years). The time that had passed between the suicide and the interviews ranged from 1 to 13 years (average 5 years), and among those lost were six males and four females.

### 2.3 Data collection

Data collection consisted of ten in‐depth individual interviews in a place chosen by each participant (Polit & Beck, 2016). The interview settings therefore varied from personal homes to other suitable locations. The interviews, for which written consent was obtained, were conducted by CO and MB separately, between October 2019 and January 2020. They lasted between 40 and 90 min and were digitally recorded. The aim of the interview structure was to adopt a conversational style initially, to allow the interviewee to speak naturally and freely about losing someone to suicide. In addition, the structure was designed to cover experiences both before and after the suicide. This afforded access to the individual's lifeworld and helped the participants reflect on their experience beyond the suicide act itself. It is through allowing expression of unreflected experiences that phenomenology methodology brings forth new knowledge (Dahlberg & Dahlberg, 2020).

To achieve this structure an interview guide with open‐ended main questions and follow‐up questions were used. The main questions were: *“Can you describe your experience of the time before the suicide?”* and *“How did you experience the time after the suicide?”* Follow‐up questions were, for example, *“What happened; how did you feel, think or act?”* Field notes were made during the interview on body language and expressed emotions, which later served as an addition to the transcript (Dahlberg et al., 2008; Dahlberg & Dahlberg, 2020).

### 2.4 Analysis

A qualitative phenomenological analysis is characterized by an open approach to the phenomenon that the participant describes (Dahlberg, 2006; Dahlberg et al., 2008), and the goal of a phenomenological study is to identify an essence that emerges from all data. (Dahlberg, 2006). The analysis was intended to create an understanding of the phenomenon and form meaning and context from a lifeworld perspective where time, space, body, and identity (Ashworth, 2003) create the horizon from which humans experience the world. The interviews were transcribed immediately after recording, and the transcript was written verbatim. The text was read in its entirety several times, and the recordings were listened to repeatedly to stay as close to the data as possible. Meaning‐bearing units that corresponded to the aim were sought. These units were reduced in number and formed a synthesis of the described experience (Dahlberg et al., 2008). The goal was to find a structure in the survivor's experiences that would enable the essence of the phenomenon to be formulated (Dahlberg, 2006). The core of what it is like to be a survivor of losing someone to suicide could then be captured (Dahlberg et al., 2008). When the analysis was complete, the essence emerged. The two coauthors (SH and RW), trained in phenomenological analysis, evaluated the data analysis to confirm its relevance and so enhance the credibility of the study (Dahlberg, 2006). The results are presented through a comprehensive description of the participants' experiences (essence) together with the variations that constitute this essence (Dahlberg et al, 2008).

### 2.5 Ethics

The research application Dnr 2016/343‐31 was approved by the Ethical Review Board in Linköping, Sweden. The ethical standards of the World Medical Association's Declaration of Helsinki (2013) were followed. Before the interviews, the informants received the information that participation was voluntary and that they could, at any time, withdraw from the study without any explanation. Collected data was deidentified and kept password‐protected, available only to the authors of the study.

## 3 FINDINGS

### 3.1 Essence description

In the experience of suicide loss, a variety of complementary dynamics appears: distance and emotional isolation, invisibility, feelings of unreality, unanswered questions, and struggle, as well as power and meaning‐making expressed through conceptual expressions and bodily sensations of loss and grief.

The experience of the self and the identity changes through interaction with others and their reactions to the relative's suicide. Experiences created in this relational dynamic, such as everyday activities, interactions and opportunities for dignified farewells, promote healing and provide an opportunity to regain a sense of control in the very confusing and fragmented situation of suicide loss.

The dynamic of grief arises as living “with grief” rather than “in grief.” In living “with grief”, reconciliation can be pursued – an opportunity to live on in a changed lifeworld where the question “*Why*?” in the story of the departed relative becomes forward‐looking and creates meaning. It is where the timeline of events loses its chronological bonds and instead floats free, that the survivors are able to highlight, observe, and re‐experience those events before leaving them in the past in processed form.

#### 3.1.1 When the darkness took over

Some of the survivors believed that their relative had dreams for the future and regular interests during the time before the suicide. They then became witness to how the darkness took over their relative's life, how despite being intelligent and well‐educated, the relative saw the joy of life diminish. Above all, survivors saw how their relative began to struggle to handle the demands of society, work and school, which created anxiety and suffering.

*When he felt bad, he went out and ran, and then it was good. The days before he did not even do that. (Participant 4)*

Many of the survivors knew that their relative was in a poor mental state, as the relative had repeatedly expressed a desire to die, but they only realized the seriousness afterwards. One survivor described how he had spent the night before the suicide with his relative watching a game of hockey. When the favorite team won the match, the relative expressed that he could die happy, a statement that was difficult to take seriously in the context. The survivors therefore believed that it was impossible to know in advance when the suicide would happen.

*I have asked around to so many, did you notice anything? But no, it came suddenly. And everyone I have asked said that they could not understand that he committed suicide. (Participant 1)*

Many survivors experienced that their relative had been suffering from mental illness for a long time and had made several suicide attempts. Due to the relative's depressive thoughts, the survivors found it difficult to make them believe that they were loved and valued. This created frustration and despair in the survivors. They also experienced that the relative was perceived as happy in the days before the suicide, to hide how they really felt.

*She was wearing a mask; did not show that she has felt bad probably all her life. (Participant 5)*

#### 3.1.2 When the worst has already happened

The survivors felt emotionally exhausted and experienced emotions of shock, sadness, anger, guilt and pain – emotions they would have to live with for the rest of their lives. They no longer feared anything, as the worst had already happened. They also described how the physical and mental pain evoked feelings they had never previously experienced. They felt the loss in their bodies as a physical pain, expressed as losing a part of their own bodies.

*Like losing a part of my body, an arm or a leg, to be amputated without anesthesia. (Participant 1)*

After the suicide, many of the survivors' personal identities changed. They were embraced by loneliness and a feeling that those around them did not understand their situation. They described differences between losing someone to suicide and losing someone to a somatic disease, and they perceived that they received less support after losing someone to suicide. After a while, neighbors, friends, and colleagues were no longer able to listen, and people who had previously been close to the family disappeared. Some survivors felt blamed by others, as they had been questioned about how they had not been able to predict the suicide. However, some people tried to comfort them by saying they did not have to feel guilty about what had happened. Regardless of the reactions of those around them, the survivors carried an inner guilt over not having been able to prevent the suicide and asked themselves if they in any way could have prevented it.

*We as relatives should not bear any guilt. But that's easier said than done. (Participant 8)*

Anger appeared after the suicide. Some survivors felt disappointed that practical things were not completed, leaving them with chores for which they had not previously been responsible. They experienced the suicide as selfish and meaningless, although, despite the anger, they seemed to understand why the suicide had happened after so many years of suffering and pain. Thus, some of them found relief after the suicide, feeling that the relative no longer had to suffer.

*But then it was definite, and so when the message came, it was hard, that's true, but it was still a little appeasement to know. (Participant 4)*

#### 3.1.3 Not a part of the care

Some of the survivors had difficulties in motivating the relative to seek care, because the relative did not believe that help was available. They described how they had tried, but with no success, mainly because the relative had negative experiences of previous admissions or had only been offered medical treatment in the past. Some survivors felt distanced from the health care, as they were not allowed to participate in the care of their relative. They said they had received too little information about mental illness, diagnoses, and symptoms. As a result, they did not feel listened to, even though they believed that they had valuable information, which could possibly have prevented the suicide. The waiting time to receive help was also criticized, and the survivors described how they had struggled over time to contact care providers.

*I was in the hospital every day and took her for walks because I simply felt that I had to check that they were taking care of her. (Participant 5)*

The right of adults to request confidentiality prevented some survivors from sharing their experiences with the care provider. Not knowing what was happening to the relative created frustration. Admission and discharge routines were problematic, and survivors highlighted situations where discharge took place too soon. It was also found that doctors assessed the relative in different ways.

*The admission was a struggle. To convince the doctors that she needs care. So, I did not feel that anyone took us seriously. I still think she would have lived if they had taken what we said and, above all, her seriously. (Participant 7)*

Several of the survivors did not know where to turn after being informed of the death. During holidays and weekends, it was difficult to get in touch with health care organizations, and they were asked to wait until the following weekday. Even if they received a telephone number to call for help, they were unable to make the first call. When they called, they experienced poor treatment and therefore made no further contact. The poor response from mental health care staff was described as lack of empathy, lack of information and only being given suggestions for other contact routes.

*They said she was dead and then we knew it. There was no priest, no psychologist. We got a phone number, where no one answered during the weekend. (Participant 3)*

On the other hand, the survivors experienced a good response when they sought help at the primary health care center.

#### 3.1.4 Forced to move on

During the immediate period after the suicide, the survivors felt shocked, as if they were living in a different world. This period mostly consisted of carrying out practical chores, such as dealing with housing organizations and organizing the memorial service. Survivors found this both healing and burdensome. During this time many survivors had difficulties eating and continuing to live their ordinary lives. It was common that survivors suffered with mental health problems and were on sick leave, but they still had to handle the situation from what they described as “the absolute bottom.”

*It was horrible. It's a strange feeling when, being so weak, you have to deal with and do a lot of things. (Participant 2)*

Even though several years had passed, the loss and grief returned, and the survivors believed it always would. At times they fell back into the black hole, but each time it became easier to find the way back. Eventually they started to feel grief and had to deal with many emotions. However, even if the grief was constantly present, it changed over time. Being forced to live with grief instead of living in grief was perceived as the goal. A quick return to everyday life was perceived as healing. Getting started with training and exercise and talking to friends or support groups of other survivors was found helpful.

*A duty; you have to do what you have to do. Monday I was away, Tuesday I worked, and I have worked all the time since then. The job is the most normal place. I functioned there; then you could forget it for a little while. (Participant 10)*

Eventually the survivors felt they either had to move on with their lives or not. The potential for moving on was improved when they had the opportunity to say goodbye. Attending funerals and memorial services was valuable because they felt the relative had a dignified end. Some of the survivors found support organizations helpful. These organizations were available around the clock, and many survivors called them for support. The survivors described how they afterwards felt compelled to do something for others in the same situation.

*I joined a support group… The longer I was there, the more I could do for others. Because he should not have died in vain. (Participant 9)*

## 4 DISCUSSION

The essence of our study was that survivors of a relative lost due to suicide described how they were forced to witness as darkness took over their relative's lifeworld. During the period before and after the suicide, the survivors felt that they were barred from having a role in the relative's care. They felt forced to move on even though the worst had happened. In this study, survivors shed light on conceptual expressions of the time before the suicide as: *“how darkness took over”* the relative's lifeworld and that their relative constructed *“a mask”* that made it difficult for them to predict the suicide. The darkness changed their relative's personality and caused them to lose interest in activities they had previously enjoyed. Survivors carried the burden of witnessing this change and felt powerless at not being able to prevent this negative spiral. Despite various observations, not all the survivors understood how bad the situation was until after the suicide. Some survivors noticed their relative's deteriorating mood before the suicide, while others did not. Among those not prepared for the suicide, being told of the death often comes as a shock (Groth et al., 2018).

Depressed people often have initial thoughts of hopelessness that eventually result in suicidal thoughts. These thoughts of hopelessness stem from the individual's feeling of pessimism, guilt and failure to achieve their dreams and goals in life (Joiner et al., 2001). However, survivors in this study described how their relatives had jobs, good financial situations and dreams of the future that were then inhibited by the darkness that plunged them into feelings of failure. This is the opposite of what Groth et al. (2018) state, namely that if a person has interests and a good financial situation, they will think less about suicide and make fewer suicide attempts. This contradiction highlights how important it is to understand and be aware of dynamic subjective factors such as “darkness,” and their implication for a specific individual, to understand how far external factors such as jobs and networks, often viewed as protective factors, truly serve as such for the individual. This highlights the need for healthcare personnel to have both an awareness of, interest in and understanding of implicit expressions to be able to give personalized care and do effective assessments of a suicidal progression (Hultsjö et al., 2018; Rytterström et al., 2020; Juel et al., 2021).

Experiencing a suicide affects both survivors and health care professionals deeply and raises the question of how suicide can be understood (Rytterström et al., 2020). Elaborating on the question of meaning can help the caregiver to be aware of the dynamics within a suicidal process and to be sensitive to them during nursing care and evaluation of the progress of a suicidal state. The “mask” that survivors in this study said their relative exhibited before the suicide made it difficult for them to predict the suicide. When the direction of death is chosen it is not uncommon for a suicidal person to hide this to have the opportunity to commit suicide (Ringel, 1976; Leenaars et al., 2020; Törnblom et al., 2013).

Survivors stated that the worst had already happened, and they felt left behind in their feelings of sadness, anger and frustration. However, although they were angry and disappointed, they seemed to understand why the suicide happened, and some found relief after the suicide, as the relative no longer had to suffer. It is common to feel disappointed and angry after losing a relative to suicide (Maple et al., 2018; Asare‐Doku et al., 2017), but to feel relief is also understandable when a relative is free from suffering (Asare‐Doku et al., 2017; Cerel et al., 2014). This feeling of relief can also be experienced by mental health care staff (Hultsjö et al., 2018) and can be explained by the empathy for the victim that developed as they tried to deal with the feelings of darkness and unworthiness in their lives. It may be important for health care professionals to be aware of such implicit dynamics, as the experience of relief, as well as addressing and actively asking about implicit dynamics during contact with relatives to evaluate the progression of a suicidal process (Berman & Silverman, 2014). Although interventions to prevent suicide occur in most countries, they neither address the aftermath of suicide, nor provide resources for suicide survivors (World Health Organization, 2021; Public Health Agency, 2020).

In this study, it emerged that the survivors felt abandoned by those around them after a relative's suicide. They felt guilt for not being able to prevent the suicide. Guilt and shame have been shown to cause people to hide the fact that suicide was the cause of death (Cerel et al., 2008), and these feelings may function as a barrier to seeking help (Nic an Fhailí et al., 2016). The stigma surrounding suicide needs further attention, even if the attitude toward talking about suicide is more open today than in previous decades Silvén Hagström (2017).

It was not only the lack of support from those around them that was mentioned but also the lack of access to health care. Survivors criticized how the care authorities dealt with their fears in the period before the suicide and most of them said they had tried to get in touch with health professionals on several occasions when they feared their relative was at risk of suicide without being taken seriously. Unfortunately, the historical culture in psychiatry can imply that relatives are the “cause” of various conditions, thus there might be a certain built‐in skepticism about the involvement of relatives (Rowe, 2012).

It has been demonstrated that survivors who receive support at an early stage after a suicide show lower morbidity (Cerel & Campbell, 2008). Yet, survivors are also shown to feel sidelined and barred from having a role in their relative's health care (Andershed et al., 2017). The majority of survivors in this study did not know where to turn for support. Previous studies have shown that survivors have negative feelings about being alone in their search for support (Nic an Fhailí et al., 2016; Shallcross Lam, 2014). However, some of the participants experienced good support from the primary health care providers, and early support such as crisis intervention is needed to help survivors further (Cerel et al., 2014).

The finding that several participants felt that primary care was better able to handle support for survivors than specialist psychiatry is interesting. Primary care deals with an unsorted and varied patient flow (Björkman & Salzmann‐Erikson, 2018, 2019), and health care professionals who work in primary care find it difficult to meet the needs of patients with mental illness within an organization, that is, not designed for their care. It is felt that these patients are hard to accommodate due to lack of time, staff and competence (Janlöv et al., 2017; Obando Medina et al.,  2014). Despite these challenges, the support of primary care is valued by the survivors. The whole situation would benefit from greater knowledge of how support for survivors should be designed (Maple et al. 2018), and highlighting the perspective of the survivors would be a good place to start.

There was a frustration about being forced to move on and go through grief, and feelings of powerlessness when it was not possible to change what had already happened. Survivors lost their hope in life and suffered mental illness and psychosomatic symptoms themselves. This is in line with previous research describing an increased risk of developing complicated grief Levi‐Belz and Aisenberg (2021) with high rates of anxiety and depression, as well as suicidal ideation and actions among survivors (Hunt & Hertlein, 2015; Levi‐Belz and Gilo, 2020; Oexle & Sheehan, 2020).

The survivors described how they felt isolated with their story and avoided telling others to avoid being judged themselves. An important part of the grieving process is to talk about the event (Peters et al., 2016b). Thus, it is important that survivors tell their story to reduce the risk of developing mental illness. Shields et al. (2019) suggest that support groups may help fill the void when survivors lack family or social support. It is also important for professionals who meet survivors to take the time to listen (Nilsson et al., 2017).

Health care professionals must try their best to prevent suicide and support survivors in a professional manner. One suggestion could be to implement postvention support for those who are grieving, as good postvention support can help people to grieve and recover (Groth et al., 2018). Adapting to grief is a dynamic and fluctuating process. Previous studies illustrate that a traumatic loss from suicide may also present new pathways to personal growth, stronger relationships and a greater appreciation for life (Ross et al., 2018) and that viewing the deceased body after suicide can have a healing but also preventive potential for survivors (Omerov et al., 2014). This was also shown in this study where survivors had the opportunity to say goodbye and to feel that their relative had a dignified end.

The trustworthiness of the study is shown by how well the themes cover the content of what emerged in the participants' stories, and that relevant data are included and irrelevant data excluded (Polit & Beck, 2016). To strengthen the credibility of the results, the themes are presented together with quotes. To make it possible for the reader to decide whether the results are transferable to a similar context, the environment in which the data collection took place and the analysis process are shown (Patton, 2015). One possible risk associated with recruiting all participants through the Swedish Association for Suicide Prevention and Survivor Support (SPES) is that they may have talked a lot about the suicide of their loved ones with each other. In this way their perceptions may have been colored by each other's experiences. However, the content of the interviews was related to the uniqueness of each lost person. At the same time, this was a pragmatic way of recruiting participants with experience of the studied phenomenon. In qualitative studies, the depth of the data is often more important than the number of people interviewed Malterud et al. (2016), and during the analysis we noticed that the same content was repeated, which enhances the trustworthiness (Patton, 2015). Nine of the 10 participants were women, and it would have been better if more men had participated. However, 70% of SPES members are women, which may be one reason behind the gender imbalance The Swedish Association for Suicide Prevention and Survivor Support SPES (2021). At the same, two‐thirds of the deceased were men, which reflects the gender ratio of the total population of people who have committed suicide (Public Health Agency of Sweden, 2020).

Interviewing survivors who have lost a relative to suicide has meant that the authors have had to reflect on ethical aspects throughout the study process. To create a feeling of security, the participants chose the place for the interviews, with most interviews conducted in their own homes. According to Dahlberg et al. (2008), an interview in one's own home creates a feeling of security and comfort, for both the interviewer and the participant. The authors were aware that the subject is sensitive and that it could provoke many reactions. Therefore, the participants were given the opportunity to take breaks.

Dahlberg et al. (2008) believe that conversations about emotional topics can provide the opportunity for the participant to work with emotions associated with previous experiences. After the interviews, the authors chose to follow up the participants with contact by telephone in order not to leave them with thoughts and feelings that might cause suffering (Polit & Beck, 2016). Dahlberg et al. (2008) emphasize the importance of the authors' responsibility to provide support for participants. During the follow‐up conversations, gratitude was expressed by the participants for having been heard, as they had long felt excluded from those in their immediate environment.

## 5 CONCLUSION

Psychiatric care could benefit from looking at the experiences of survivors of suicide loss in order understand suicidal progression in more depth. Survivors' experiences in the time leading up to and after the suicide are similar to professionals' experiences of losing a patient to suicide. An awareness of implicit dynamics, such as “a feeling of darkness taking over,” “a sense of relief,” or “putting on a mask” could be important for evaluating both suicidal progress and preventive care. Nurses have an important preventive role in meeting suffering survivors to support their grieving process. Nurses and other healthcare professionals therefore need to understand and be able to confirm the implicit dynamics and forbidden feelings as described in this study. The survivors found it helpful to talk with others in similar situations, which shows that information about support groups can be helpful.

### 5.1 Implications for nursing practice

- Understanding and exploring implicit dynamics, such as “a feeling of darkness taking over,” “a sense of relief,” or “putting on a mask” could be important for developing person‐centered suicide care.
- Survivors need to be offered support to deal with the crisis that arises after suicide and to prevent the development of mental illness. This support should also confirm forbidden feelings of relief.
- Survivors find it helpful to share their stories with other survivors and need information about how to get in touch with support groups.

## CONFLICT OF INTERESTS

The authors declare no conflict of interest.

## Data Availability

Data are available on request from the authors.

## References

[ppc13049-bib-0001] Andershed, B. , Ewertzon, M. , & Johansson, A. (2017). An isolated involvement in mental health care: Experiences of parents of young adults. Journal of Clinical Nursing, 26(7,8), 1053–1065. 10.1111/jocn.13560 27570938

[ppc13049-bib-0002] Asare‐Doku, W. , Osafo, J. , & Akotia, C. S. (2017). The experiences of attempt survivor families and how they cope after a suicide attempt in Ghana: A qualitative study. BMC Psychiatry, 11(1), 178. 10.1186/s12888-017-1336-9 PMC5426021 28490324

[ppc13049-bib-0003] Ashworth, P. (2003). The phenomenology of the lifeworld and social psychology. Social Psychological Review, 5(1), 18–34.

[ppc13049-bib-0004] Berman, A. L. , & Silverman, M. M. (2014). Suicide risk assessment and risk formulation part II: Suicide risk formulation and the determination of levels of risk. Suicide and Life‐Threatening Behavior, 44(4), 432–443. 24286521 10.1111/sltb.12067

[ppc13049-bib-0005] Björkman, A. , & Salzmann‐Erikson, M. (2018). When all other doors are closed: Telenurses' experiences of encountering care seekers with mental illnesses. International journal of mental health nursing, 27(5), 1392–1400. 10.1111/inm.12438 29383820

[ppc13049-bib-0006] Björkman, A. , & Salzmann‐Erikson, M. (2019). Giving advice to callers with mental illness: Adaptation among telenurses at Swedish Healthcare Direct. International Journal of Qualitative Studies on Health and Well‐Being, 14(1), 1–9. 10.1080/17482631.2019.1633174 PMC6598523 31242817

[ppc13049-bib-0007] Cerel, J. , & Campbell, F. (2008). Suicide survivors seeking mental health services: A preliminary examination of the role of an active postvention model. Suicide and Life‐Threatening Behavior, 38(1), 30–34. 10.1521/suli.2008.38.1.30 18355106

[ppc13049-bib-0008] Cerel, J. , Jordan, J. R. , & Duberstein, P. R. (2008). The impact of suicide on the family. Crisis, 29(1), 38–44. 10.1027/0227-5910.29.1.38 18389644

[ppc13049-bib-0009] Cerel, J. , McIntosh, J. L. , Neimeyer, R. A. , Maple, M. , & Marshall, D. (2014). The continuum of “survivorship”: Definitional issues in the aftermath of suicide. Suicide and Life‐Threatening Behavior, 44(6), 591–600. 10.1111/sltb.12093 24702241

[ppc13049-bib-0010] Chapple, A. , Ziebland, S. , & Hawton, K. (2015). Taboo and the different death? Perceptions of those bereaved by suicide or other traumatic death. Sociology of Health and Illness, 37(4), 610–625. 10.1111/1467-9566.12224 25683372

[ppc13049-bib-0011] Dahlberg, H. , & Dahlberg, K. (2020). Open and reflective lifeworld research: A third way. Qualitative Inquiry, 26(5), 458–464. 10.1177/1077800419836696

[ppc13049-bib-0012] Dahlberg, K. (2006). The essence of essence: The search for meaning structures in phenomenological analysis of lifeworld phenomena. Journal of Health and Well‐being, 1, 11–19. 10.1080/17482620500478405

[ppc13049-bib-0013] Dahlberg, K. , Dahlberg, H. , & Nyström, M. (2008). Reflective lifeworld research. Studentlitteratur AB.

[ppc13049-bib-0014] Dransart, D. A. C. (2017). Reclaiming and reshaping life: Patterns of reconstruction after the suicide of a loved one. Qualitative Health Research, 27(7), 994–1005. 10.1177/1049732316637590 27055497

[ppc13049-bib-0015] Eckholdt, L. , Watson, L. , & O'Connor, M. (2018). Prolonged grief reactions after old age spousal loss and centrality of the loss in post‐loss identity. Journal of Affective Disorders, 227, 338–344. 10.1016/j.jad.2017.11.010 29136603

[ppc13049-bib-0016] Entilli, L. , Ross, V. , De Leo, D. , Cipolletta, S. , & Kõlves, K. (2021). Experiences of parental suicide‐bereavement: A longitudinal qualitative analysis over two years. International Journal of Environmental Research and Public Health, 18(2). 10.3390/ijerph18020564 PMC7826588 33440875

[ppc13049-bib-0017] Erlangsen, A. , Runeson, B. , Bolton, J. , Wilcox, H. , Forman, J. , Krogh, J. , Shear, M. , Nordentoft, M. , & Conwell, Y. (2017). Association between spousal suicide and mental, physical, and social health outcomes: A longitudinal and nationwide register‐based study. JAMA Psychiatry, 74(5), 456–464. 10.1001/jamapsychiatry.2017.0226 28329305 PMC5470398

[ppc13049-bib-0018] Feigelman, W. , Cerel, J. , McIntosh, J. L. , Brent, D. , & Gutin, N. (2018). Suicide exposures and bereavement among American adults: Evidence from the 2016 General Social Survey. Journal of Affective Disorders, 227, 1–6. 10.1016/j.jad.2017.09.056 29045914

[ppc13049-bib-0019] Groth, C. J. , Anthony, M. , & Gash, J. (2018). The aftermath of suicide: A qualitative study with Guyanese families. Archives of Psychiatric Nursing, 32(3), 469–474. 10.1016/j.apnu.2018.01.007 29784232

[ppc13049-bib-0020] Health Care Act . (2017). 30. Stockholm, Sweden.

[ppc13049-bib-0021] Hultsjö, S. , Wärdig, R. , Rytterström, P. (2018). The borderline between life and death: Mental healthcare professionals' experience of why patients commit suicide during ongoing care. Journal of Clinical Nursing, 28(9,10). 10.1111/jocn.14754 30589485

[ppc13049-bib-0022] Hunt, Q. , & Hertlein, K. (2015). Conceptualizing suicide bereavement from an attachment lens. The American Journal of Family Therapy, 43(1), 16–27. 10.1080/01926187.2014.975651

[ppc13049-bib-0023] Janlöv, A. C. , Johansson, L. , & Clausson, E. (2017). Mental ill‐health among adult patients at healthcare centres in Sweden: District nurses' experiences. Scandinavian Journal of Caring Sciences, 32(2), 987–996. 10.1111/scs.12540 29131370

[ppc13049-bib-0024] Joiner, T. E. , Steer, R. A. , Abramson, L. Y. , Alloy, L. B. , Metalsky, G. I. , & Schmidt, N. B. (2001). Hopelessness depression as a distinct dimension of depressive symptoms among clinical and non‐clinical samples. Behaviour Research and Therapy, 39(5), 523–536. 10.1016/s0005-7967(00)00024-3 11341249

[ppc13049-bib-0025] Juel, A. , Berring, L. L. , Hybholt, L. , Erlangsen, A. , Larsen, E. R. , & Buus, N. (2021). Relatives' experiences of providing care for individuals with suicidal behaviour conceptualized as a moral career: A meta-ethnographic study. International Journal of Nursing Studies, 113, N.PAG. 10.1016/j.ijnurstu.2020.103793 33161331

[ppc13049-bib-0026] Kõlves, K. , Zhao, Q. , Ross, V. , Hawgood, J. , Spence, S. H. , & de Leo, D. (2019). Suicide and sudden death bereavement in Australia: A longitudinal study of family members over 2 years after death. Australian and New Zealand Journal of Psychiatry, 54(1), 89–98. 10.1177/0004867419882490 31647307

[ppc13049-bib-0027] Lee, E. , Kim, S. W. , Kim, J. J. , & Enright, R. D. (2017). Family conflict and forgiveness among survivors of suicide. Journal of Loss and Trauma, 22(8), 689–697. 10.1080/15325024.2017.1388344

[ppc13049-bib-0028] Leenaars, A. A. , Dieserud, G. , & Wenckstern, S. (2020). The mask of suicide. Archives Of Suicide Research, 30, 1–22. 10.1080/13811118.2020.1851832 33256543

[ppc13049-bib-0029] Levi‐Belz, Y. , & Lev‐Ari, L. (2019a). Is there anybody out there? Attachment style and interpersonal facilitators as protective factors against complicated grief among suicide‐loss survivors. Journal of Nervous and Mental Disease, 207(3), 131–136. 10.1097/nmd.0000000000000940 30720603

[ppc13049-bib-0030] Levi‐Belz, Y. , & Lev‐Ari, L. (2019b). “Let's talk about it”: The moderating role of self‐disclosure on complicated grief over time among suicide survivors. Int Journal of Environmental Research and Public Health, 16(19), 3740. 10.3390/ijerph16193740 31590225 PMC6801618

[ppc13049-bib-0031] Levi‐Belz, Y. , & Gilo, T. (2020). Emotional distress among suicide survivors: The moderating role of self‐forgiveness. Frontiers in Psychiatry, 11, 341. 10.3389/fpsyt.2020.00341 32390889 PMC7190787

[ppc13049-bib-0032] Levi‐Belz, Y. , & Aisenberg, D. (2021). Interpersonal predictors of suicide ideation and complicated‐grief trajectories among suicide bereaved individuals: A four‐year longitudinal study. Journal of Affective Disorders, 282, 1030–1035. 10.1016/j.jad.2021.01.006 33601675

[ppc13049-bib-0033] Obando Medina, C. , Kullgren, G. , & Dahlblom, K. (2014). A qualitative study on primary health care professionals' perceptions of mental health, suicidal problems and help‐seeking among young people in Nicaragua. BMC Family Practice, 2(15), 129. 10.1186/1471-2296-15-129 PMC4112650 24989871

[ppc13049-bib-0034] Malterud, K. , Siersma, V. D. , & Guassora, A. D. (2016). Sample size in qualitative interview studies: Guided by information power. Qualitative Health Research, 26(13), 1753–1760. 10.1177/1049732315617444 26613970

[ppc13049-bib-0035] Maple, M. , McKay, K. , Hess, N. , Wayland, S. , & Pearce, T. (2018). Providing support following exposure to suicide: A mixed method study. Health and social care, 27(4), 965–972. 10.1111/hsc.12713 30680822

[ppc13049-bib-0036] Maple, M. , Plummer, D. , Edwards, H. , & Minichiello, V. (2007). The effects of preparedness for suicide following the death of a young adult child. Suicide and Life‐Threatening Behavior, 37(2), 127–134. 10.1521/suli.2007.37.2.127 17521266

[ppc13049-bib-0037] McMenamy, J. , Jordan, J. , & Mitchell, A. (2008). What do suicide survivors tell us they need? Results of a pilot study. Suicide and Life‐Threatening Behavior, 38(4), 375–89. 10.1521/suli.2008.38.4.375 18724786

[ppc13049-bib-0038] Mogensen, H. , Möller, J. , Hultin, H. , & Mittendorfer‐Rutz, E. (2016). Death of a close relative and the risk of suicide in Sweden: A large‐scale register‐based case‐crossover study. PLoS One, 11(10). 10.1371/journal.pone.0164274 PMC5058490 27727324

[ppc13049-bib-0039] Nic an Fhailí, M. , Flynn, N. J. , Dowling, S. , Fhailí, M. N. A. , Flynn, N. , Dowling, S. , & Nic an Fhailí, M. (2016). Experiences of suicide bereavement: A qualitative study exploring the role of the GP. British Journal of General Practice, 66(643), e92–e98. 10.3399/bjgp16X683413 PMC4723220 26823270

[ppc13049-bib-0040] Nilsson, C. , Bremer, A. , & Svantesson, M. (2017). Responsibility and compassion in prehospital support to survivors of suicide victim: Professionals' experiences. International emergency nursing, 35, 37–42. 10.1016/j.ienj.2017.06.004 28687433

[ppc13049-bib-0041] Oexle, N. , & Sheehan, L. (2020). Perceived social support and mental health after suicide loss. Crisis, 41(1), 65–69. 10.1027/0227-5910/a000594 31030548

[ppc13049-bib-0042] Omerov, P. , Steineck, G. , Nyberg, T. , Runeson, B. , & Nyberg, U. (2014). Viewing the body after bereavement due to suicide: A population‐based survey in Sweden. PLoS One, 9(7), e101799. 10.1371/journal.pone.0101799 24999660 PMC4085007

[ppc13049-bib-0043] Patton, M. Q. (2015). Qualitative research and evaluation methods (4th ed.). Sage.

[ppc13049-bib-0044] Peters, K. , Cunningham, C. , Murphy, G. , & Jackson, D. (2016a). Helpful and unhelpful responses after suicide: Experiences of bereaved family members. International Journal of Mental Health Nursing, 25(5), 418–425. 10.1111/inm.1222418-425 27037948

[ppc13049-bib-0045] Peters, K. , Cunningham, C. , Murphy, G. , & Jackson, D. (2016b). ‘People look down on you when you tell them how he died': Qualitative insights into stigma as experienced by suicide survivors. International Journal of Mental Health Nursing, 25(3), 251–257. 10.1111/inm.12210.25.251-257 26889754

[ppc13049-bib-0046] Pitman, A. L. , Osborn, D. P. J. , Rantell, K. , & King, M. B. (2016). The stigma perceived by people bereaved by suicide and other sudden deaths: A cross‐sectional UK study of 3432 bereaved adults. Journal of Psychosomatic Research, 87, 22–29. 10.1016/j.jpsychores.2016.05.009 27411748 PMC4988532

[ppc13049-bib-0047] Polit, D. F. , & Beck, C. T. (2016). Nursing research: Generating and assessing evidence for nursing practice (10th ed.). Lippincott, Williams and Wilkins.

[ppc13049-bib-0048] Public Health Agency of Sweden . (2020). Suicide and suicide prevention in Sweden. https://www.folkhalsomyndigheten.se/the-public-health-agency-of-sweden/living-conditions-and-lifestyle/suicide-prevention/

[ppc13049-bib-0049] Ringel, E. (1976). The presuicidal syndrome. Suicide and Life‐Threatening Behavior, 6(3), 131–149. 996916

[ppc13049-bib-0050] Ross, V. , Kõlves, K. , Kunde, L , & De Leo, D. (2018). Parents' experiences of suicide‐bereavement: A qualitative study at 6 and 12 months after loss. International Journal of Environmental Research and Public Health, 15(4), 618. 10.3390/ijerph15040618 29597297 PMC5923660

[ppc13049-bib-0051] Rowe, J. (2012). Great expectations: A systematic review of the literature on the role of family carers in severe mental illness, and their relationships and engagement with professionals. Journal of Psychiatric and Mental Health Nursing, 19(1), 70–82. 10.1111/j.1365-2850.2011.01756.x 22070436

[ppc13049-bib-0052] Shields, C. , Russo, K. , & Kavanagh, M. (2019). Angels of courage: The experiences of mothers who have been bereaved by suicide. Omega (Westport), 80(2), 175–201. 10.1177/0030222817725180 28882098

[ppc13049-bib-0053] Rytterström, P. , Ovox, S. , Wärdig, R. , & Hultsjö, S. (2020). Impact of suicide on health professionals in psychiatric care: Mental healthcare professionals' perceptions of suicide during ongoing psychiatric care and its impacts on their continued care work. International Journal of Mental Health Nursing, 29(5), 982–991. 10.1111/inm.12738 32419316

[ppc13049-bib-0054] Sellin, L. , Asp, M. , Kumlin, T. , Wallsten, T. , & Wiklund Gustin, L. (2017). To be present, share and nurture: A lifeworld phenomenological study of relatives' participation in the suicidal person's recovery. International Journal of Qualitative Studies on Health & Well‐Being, 12(1), N.PAG. 10.1080/17482631.2017.1287985 PMC5345596 28245364

[ppc13049-bib-0055] Shallcross Lam, W. (2014). In their own words: Perceived experiences and family functioning of suicide survivors before and after suicide loss (Dissertation). Philadelphia: Philadelphia College of Osteopathic Medicine. https://digitalcommons.pcom.edu/cgi/viewcontent.cgi?article=1291%26context=psychology_dissertations

[ppc13049-bib-0056] Shear, M. K. (2015). Complicated grief. New England Journal of Medicine, 372, 153–160. 10.1056/NEJMcp1315618 25564898

[ppc13049-bib-0057] Silvén Hagström, A. (2017). Breaking the silence: Parentally suicide‐bereaved youths' self‐disclosure on the internet and the social responses of others related to stigma. Journal of Youth Studies, 20(8), 1077–1092. 10.1080/13676261.2017.1307330

[ppc13049-bib-0058] Skundberg‐Kletthagen, H. , Wangensteen, S. , Hall‐Lord, M.‐L. , & Hedelin, B. (2014). Relatives of patients with depression: Experiences of everyday life. Scandinavian Journal of Caring Sciences, 28(3), 564–571. 24111931 10.1111/scs.12082

[ppc13049-bib-0059] Sugrue, J. L. , McGilloway, S. , & Keegan, O. (2014). The experiences of mothers bereaved by suicide: An exploratory study. Death Studies, 38(2), 118–124. 10.1080/07481187.2012.738765 24517710

[ppc13049-bib-0060] Sveen, C. A. , & Walby, F. A. (2008). Suicide survivors' mental health and grief reactions: A systematic review of controlled studies. Suicide and Life‐Threatening Behavior, 38(1), 13–29. 10.1521/suli.2008.38.1.13 18355105

[ppc13049-bib-0061] The Swedish Association for Suicide Prevention and Survivor Support (SPES) . (2021). https://spes.se/

[ppc13049-bib-0062] Törnblom, A. , Werbart, A. , & Rydelius, P.‐A. (2013). Shame behind the masks: The parents' perspective on their sons' suicide. Archives of Suicide Research, 17(3), 242–261. 10.1080/13811118.2013.805644 23889574

[ppc13049-bib-0063] Walker, R. (2017). After suicide: Coming together in kindness and support. Death Studies, 41(10), 635–638. 10.1080/07481187.2017.1335549 28548614

[ppc13049-bib-0064] World Health Organization . (2019). Suicide. https://www.who.int/news-room/fact-sheets/detail/suicide

[ppc13049-bib-0065] World Health Organization . (2021). Suicide prevention toolkit for engaging communities. https://www.who.int/mental_health/suicide-prevention/engaging_communities_toolkit/en/

